# Homelessness and Emergency Department Use: Wait Time Disparities Across Triage Acuity Levels

**DOI:** 10.7759/cureus.49520

**Published:** 2023-11-27

**Authors:** Swarna S Weerasinghe, Samuel G Campbell

**Affiliations:** 1 Department of Community Health and Epidemiology, Dalhousie University, Halifax, CAN; 2 Department of Emergency Medicine, Dalhousie University, Halifax, CAN

**Keywords:** canadian triage acuity scale-based ed visits, wait time benchmarks, disparities across demographic subgroups, wait time, homelessness

## Abstract

Introduction

Certain patient groups perceive specific barriers to accessing primary care, resulting in increased emergency department (ED) use for non-emergency conditions. There is evidence coming from other countries that homeless people are treated differently in accessing emergency services. Examination of ED wait time by demographic characteristics provides pertinent information to identify subgroups that are more subject to the consequences or causes of access block and delayed treatment.

Methods

We analyzed five years of Emergency Department Information System (EDIS) visit records of the largest tertiary care center in Atlantic Canada. The wait time from triage to seeing a physician was the outcome, housing status was the main exposure, and age and gender were the predictors. Quantile regressions were carried out to identify the influence of homeless visits in meeting the Canadian Association of Emergency Physicians (CAEP) wait time benchmarks for each Canadian Triage and Acuity Scale (CTAS) level. The classification and regression tree method was used to quantify and classify the demographic subgroups contributing to wait time disparities across CTAS levels.

Results

Homeless visit median wait times that exceeded the three-hour CAEP benchmark were significantly longer for urgent (by 40 minutes, CI: 25-55), semi-urgent (by 31 minutes, CI: 17-45), and non-urgent (by 57 minutes, CI: 25-89) than acuity level-matched domiciled visit wait times. At the 50th percentile, one-hour benchmark homeless triaged as semi-urgent waited (median=20 minutes, CI: 12-28) longer, and no other triage-level differences were found at this benchmark. Homeless emergent-level visits that exceeded the three-hour benchmark were 28 minutes, on average, shorter than domiciled patients of the same acuity level. Homeless females above 40 stayed the longest for non-urgent care (mean=173 minutes), 82 minutes longer (p=0.0001) than age-gender-acuity level-matched domiciled patients. Homelessness was the most prominent ED wait time classifier for non-urgent, ED visits. Overall, homeless patients triaged as CTAS-5 waited 30 minutes longer (p=0.0001) than domiciled patients triaged as CTAS-5. Homeless male 16-20-year-olds waited the shortest time of 72 minutes.

Conclusion

Homelessness-related wait time disparities exist in the low acuity non-urgent-level ED visits more than in the other levels, supporting the theory that lack of primary care access is a driver of ED use in this group. Our acuity level analysis supports that homeless people of a certain age (older) and gender groups (female) wait longer than their age-gender-matched domiciled patients to be seen by a physician in low acuity level presentations. Given the pattern of the homeless being seen earlier or statistically similar in emergent-level visits compared to matched domiciled patients and that 16-20-year-old homeless males were seen on average within 72 minutes (the shortest mean wait time reported for the triage level CTAS-5), we decline the notion of discrimination at the study site ED. If homeless patients' non-urgent needs were met elsewhere, pressure on the ED to meet benchmarks might be reduced.

## Introduction

Emergency department (ED) overcrowding is a worldwide problem, no less in Canada with a shortage of inpatient beds and limited access to primary care for newcomers, including international and interprovincial migrants and the homeless [1]. Certain high-risk groups perceive specific barriers to accessing healthcare. Qualitative research involving Calgary's homeless population identified fear of authority and unwelcoming atmospheres as perceived barriers to accessing primary care [2] and noted this resulted in increased ED access for non-urgent conditions. ED overcrowding is defined as the situation that arises when the demand to provide services within an appropriate time frame exceeds the capacity [3]. The Canadian Association of Emergency Physicians (CAEP) has stated that ED overcrowding is not caused by inappropriate use of ED for low acuity level conditions but by taking a long wait time to be admitted as an inpatient, denoted by "access block" [3,4]. CAEP has specified appropriate time frames based on acuity levels. In that regard, examination of ED wait time patterns associated with different demographic characteristics provides pertinent information to identify subgroups that are more subject to the consequences or causes of access block. Canadian researchers have shown that homeless people are heavy users of emergency services [5]. US-based research has shown that the homeless wait longer than domiciled patients to receive ED care [6].

Long wait times for ED care are significantly associated with patient dissatisfaction and are used as a measure of ED quality [7]. Long wait times for high acuity conditions are associated with an increase in mortality among ED patients. The mortality rate of Canadian street youth is 11 times the expected rate of age-gender-matched off-street individuals [8]. A study conducted in the United States that compared ED wait times of homeless to domiciled patients found that when categorized by emergent and urgent acuity levels, homeless people waited longer, although wait times were statistically similar for immediate- and non-urgent-level visits [6]. It is unclear whether this would be similar in the Canadian context of universal healthcare coverage.

Homelessness is defined as lacking stable, permanent, and appropriate housing, including unsheltered, emergency-sheltered, and provisionally accommodated individuals [9]. In Canada, an estimated 235,000 individuals experience homelessness each year, with 35,000 experiencing homelessness on any given night [10]. The reasons for the homeless crisis in Canada, as indicated in the report, were lack of affordable housing in Canada and reduced affordability due to job loss and lack of permanent jobs [10]. The majority (73%) of homeless are males and 19% are youth [10]. Patients suffering from homelessness are disproportionate in their need for emergency healthcare services. In 2018, 45% of homeless people in Halifax reported being physically assaulted, requiring immediate ED care, and 82% of females and 66% of males had used emergency healthcare [11,12]. The city of Halifax, Nova Scotia, has the fifth largest homeless population in Canada with an estimated 586 homeless people on any given night (point-in-time counts) [10]. In 2011, 1973 people stayed in shelters in Halifax, with homeless numbers growing each year [13]. The study site, Charles V. Keating Emergency and Trauma Centre (noted as Halifax Infirmary or HI, herein), is the largest ED in Atlantic Canada, with approximately 200 ED visits per day [14]. Although homeless ED visits represent less than 2% of the general population, they frequently have more complex needs and require more resources [15]. A study on homeless healthcare utilization in Ontario revealed homeless patients had an 8.5 times higher rate of ED utilization than age-gender-matched low-income domiciled controls in the province, due to increased ambulatory care encounters [5], but acuity level-based wait time disparities remain under-reported. The scope of this study is to provide insight into ED wait time patterns across demographically diverse groups and to identify subgroups of homeless adults at risk for prolonged wait times to be seen by an ED physician.

Rationale

Anecdotal and research-based evidence from other ED have suggested wait time disparities across demographic statuses [6,16]. There is no research-based evidence on ED use wait time patterns by homeless compared to domiciled patients in the study city of Halifax, Canada. Homelessness is a growing problem in Canada, and statistics based on Ontario ED visits show a rapid annual increase in homeless visits [15]. Another Canadian city-based qualitative study has noted "clinical bias" towards homeless ED patients, suggesting discrimination against those with mental illnesses, chronic pain, and addictions [17]. This article provides a quantitative investigation of demographic patterns of ED use across triage acuity levels, information vital to understanding the potential effect of the growing homeless population on ED demands.

There is compelling evidence in the Canadian literature that homeless people have more health issues than the general population [18,19]. An estimated 83% of homeless Canadians suffer from drug dependency with 93% having mental health disorders [20]. Homeless youth experience infectious diseases, pregnancy-related issues, and violence at significantly higher rates than their housed peers [8]. Homeless individuals have significantly higher mortality rates than the poorest fifth of Canadians, and the life expectancy at the age of 25 is 42 years [21]. Compared to the housed people, homeless people in Canada are twice as likely to die from violence-related trauma [10]. This under-studied population has greater emergency care needs than the domiciled population, and the system needs research-based evidence to meet their ED needs.

Multi-morbidity is the strongest predictor of ED use [22]. The vast majority of homeless people in Canada suffer from at least two medical conditions with the likelihood of this doubling in every five-year increment of age [12], hence increasing their potential to become heavy per capita users of healthcare services as they get older. In 2009, 47% of homeless people in Halifax reported visiting the ED at least once during the previous 24 months [11]. The research indicated higher standardized rates of ED use by the homeless and an increasing trend in high acuity ED presentations by the homeless in Halifax [23]. Only 43% of homeless individuals in Toronto and 63% in British Columbia report having a family physician or nurse practitioner [17,19], and only 9% in Alberta reported having their healthcare needs met [24]. Therefore, low acuity level ED presentations by the homeless can be considered inevitable. No Canadian studies had compared the ED wait times of homeless to domiciled people across acuity levels. Filling this gap in knowledge would allow social and healthcare planners to address specific sub-population-level determinants of health and need-based access planning and delivery.

Long ED wait times are significantly associated with patient dissatisfaction and are used as an indicator of poor-quality care [7]. Long wait times are associated with an increase in mortality among ED patients requiring admission for acute medical conditions [25]. A US study that compared ED wait times of homeless to domiciled patients found that, when categorized by acuity levels, homeless people triages to emergent and urgent waited longer, although wait times among the two groups were statistically similar for immediate- and non-urgent-level visits [6]. This research explores the issue in the context of a Canadian tertiary care ED. 

Purpose

In this study, we focus on adult (16 and above years of age) ED wait times, from the time of triage until being seen by a physician. The 2013 CAEP position paper specifies benchmarks for Canadian Triage and Acuity Scale (CTAS)-based ED wait times, indicating a median wait of one hour and a 90th percentile target wait time of three hours [25]. 

The goal of this study is to elucidate the achievement of CAEP benchmarks and identify acuity-based wait time disparities between homeless and domiciled patients for the five-year period from 2011 to 2015. Our objectives are to (i) describe median wait times of homeless and domiciled patients by demographic and CTAS levels; (ii) identify factors associated with (a) achieving the ED wait time CAEP 50th percentile, benchmark of one hour, and 90th percentile benchmark of three hours and (b) ED wait time exceeding the three-hour benchmark; and (iii) identify age-gender-housing status subgroups associated with excess wait times and contributing to wait time disparities as specified in the Canadian literature [25,26]. The findings will allow targeted healthcare planning for these specific age-gender-classified homeless groups.

## Materials and methods

The Emergency Department Information System (EDIS) data were collected from the Charles V. Keating Emergency and Trauma Centre, Halifax Infirmary (HI), Queen Elizabeth II (QEII) Health Sciences Center, Halifax, Nova Scotia, Atlantic Canada, on January 28, 2016. Our dataset contained individual-level demographics (age and gender) and shift of visits and CTAS. The inability to provide a valid residential address has been used as an indicator of homelessness by Statistics Canada [14]. We use the same indicator in the EDIS records to classify homeless visits. Wait time was defined as the time from triage to be seen by an ED physician. Those who left without being seen (LWBS) were excluded from the detailed analyses. The prescribed maximum waiting period to be seen is determined at triage by CTAS. The CTAS levels that range from 1 to 5 as indicated in Table 1 have been specified according to the Canadian literature [26]. Our analysis could not evaluate wait time differences across housing status for CTAS-1, as there were very few homeless visits in this category, which were thus excluded from further analysis.

**Table 1:** CTAS description CTAS: Canadian Triage and Acuity Scale

CTAS level	Description	Acuity level	Recommended time to physician
1	Life-threatening conditions requiring immediate aggressive interventions	Resuscitation	Immediate
2	Emergent conditions necessitating rapid medical intervention	Emergent	<15 minutes
3	Conditions that could potentially progress to a serious problem	Urgent	30 minutes
4	Conditions that relate to the potential for deterioration (120 minutes) that would benefit from intervention	Less urgent	60 minutes
5	Conditions that may be acute but non-urgent and interventions can be safely delayed	Non-urgent	120 minutes

Given that CAEP guidelines focus on wait time percentiles to compensate for right-skewed wait time distribution, our analyses focus on quantile regression, analyzing the median and the 90th percentile of wait times calculated across demographic characteristics. We carried out quantile regression by using two benchmarks, the first being the median wait time as the dependent variable in the model, gender and age as confounders, and housing status as the exposure. Quantile regression has been used in ED wait time analyses, and the model accuracy has been validated [27]. We fitted three models. The first two models were fitted to the 50th and 90th percentiles of wait times by four CTAS levels (levels 2-5) to identify the demographic predictors of wait time. The third model was fitted to the subset of those who stayed longer than three hours to elucidate the homeless contribution to lengthy wait times exceeding CAEP targets. These three quantile regressions by CTAS levels used housing status (homeless or domiciled) as the exposure binary variable and age groups, categorized as less than 20, 20-40, 40-60, and above 60, and gender (male, female) as covariates. These age cut-offs were chosen based on the initial classification tree-splitting criteria. As mentioned, the CTAS-1 level was excluded in detailed analyses due to low patient numbers and small variations in wait times across demographic characteristics.

The classification and regression tree (CART) method uses the deviance reduction criteria for splitting [28] and provides the importance of each demographic variable for the wait time disparities, using wait time variations among subgroups. Each split was determined by minimizing the error sums of squares from the analysis of variance. For example, when classifying the wait times by housing status, homeless, and domiciled, the departure of homeless weight times from the mean was summed over all homeless patients, and this quantity was added to the same of the domiciled. If the total was the lowest of all of the other classifications (across age groups or gender), housing status was considered as the dominant classifier. In a mathematical formula, the deviance as the error sum of squares equals to ∑_h_(w-w^=^)-∑_d_(w-w^=^)​, where h and d indicate the summation over all the classifiers, of homeless and domiciled, and w^=^ indicates the mean wait time for the specific group. The classification criteria continue using the same criteria across each of the already unclassified subgroups. The rpart package in R software produces mean wait times, and this is noted in the final nodes. Comparison of wait time means across demographically classified final subgroups is appropriate since they are homogeneous and normally distributed. Existing software packages do not support median classification. All analyses were conducted using R software version 4.1 [29].

## Results

Patient characteristics

Our data contained 350,111 visits, made over 1826 days, during the period of 2011-2015 to the HI ED. On average, there were 192 visits per day with a range of 99-254/day. Of those visits, 347,112 (99.1%) were domiciled visits, whereas 3081 (0.9%) were homeless visits made over 1441 days. There were significant differences between homeless and domiciled ED visits across age groups. The highest percentage of homeless visits were by those younger than 30 years of age (homeless 50.2% versus domiciled 41.4%, p<0.0001) and less by above 60-year-olds (homeless 1.3% versus domiciled 15%, p<0.0001). We also found fewer female homeless visits (homeless 23% versus 53% domiciled, p<0.0001) compared to female domiciled visits. Most homeless visits (43%) occurred between 3pm and 11pm (Table 2). ED visits that concluded without the patient being seen by an ED physician (LWBS) were more common (16%) among the homeless compared to domiciled patients (2%, p<0.0001). In terms of acuity level, 90% (versus 86% of domiciled) of homeless LWBS had been assigned to CTAS acuity levels 3-5 accounting for 17.2% (CTAS-3), 17.5% (CTAS-4), and 19% (CTAS-5). LWBS visits were excluded from further analyses. Table 2 contains the median wait time and interquartile range (IQR) in minutes.

**Table 2:** Patient visit characteristics, percent of visits, median wait times, and IQR over five years CTAS: Canadian Triage and Acuity Scale; IQR: interquartile range Significance levels (p-values) for the median wait times of homeless and domiciled patients were compared: 0.1 is noted as *, 0.05 is noted as **, and <0.01 is noted as *** which is highly significant

Characteristic (n=5-year visit total)	Homeless visits (%) n=3081	Domiciled median wait time in minutes (IQR)	Homeless median wait time in minutes (IQR)	p-value (wait time differences)
Age group				
16-20 (n=21856)	31.4	100 (58-165)	89 (47-162)	0.03**
21-40 (n=123229)	37.4	92 (54-153)	89 (48-178)	0.53
41-60 (n=98987)	30.0	85 (51-143)	90 (47-187)	0.18
Above 60 (n=106039)	1.2	89 (56-141)	58 (33-92)	0.009***
Gender				
Male (n=84695)	77.2	83 (50-139)	86 (46-167)	0.24
Female (n=84562)	22.8	99 (59-162)	106 (55-199)	0.09^*^
CTAS				
1 (n=3539)	0.8	14 (6-27)	14 (7-34)	1.0
2 (n=39159)	19.6	60 (39-94)	60 (35-105)	0.9
3 (n=63058)	40.4	119 (75-183)	112 (61-217)	0.3
4 (n=48853)	27.2	81 (49-132)	100 (51-191)	0.0001^***^
5 (n=14733)	12.0	75 (43-119)	77 (46-156)	0.8
Shift (time of the day)				
7am-3pm (n=66668)	32.0	83 (53-132)	77 (42-126)	0.005^***^
3pm-11pm (n=63027)	43.0	99 (56-168)	94 (49-193)	0.2
11pm-7am (n=39647)	25.0	105 (57-172)	121 (54-224)	0.04^**^
Season				
Fall	26.5	88 (53-146)	84 (45-168)	0.3
Spring	24.5	86 (52-141)	80 (44-146)	0.04*
Summer	27.4	93 (56-153)	96 (52-189)	0.4
Winter	21.6	99 (58-166)	103 (52-204)	0.7
Left without being seen	84	-	-	-

Descriptive statistics of median wait times

**Figure 1:**
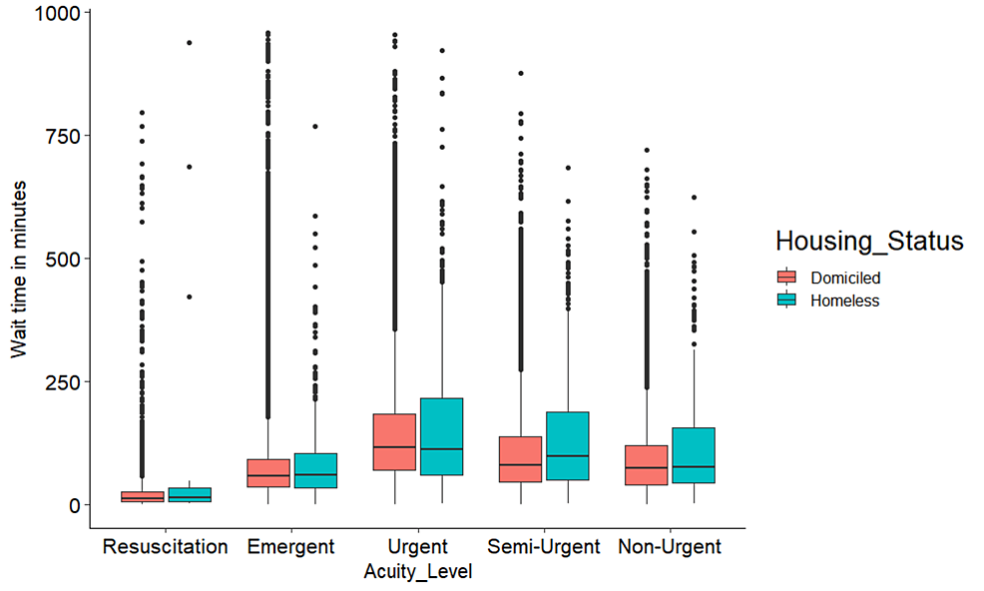
Wait time across acuity levels

The wait time distribution (Figure 1) is skewed to the right (long tail upward) in both housing status groups. The disparities between the two housing groups exist in the upper quartile of the distribution. The median indicates the wait time at the 50% of the visits (horizontal bar in Figure 1 box plot). The median wait time was significantly shorter (p=0.009) in the homeless subset of above 60-year-olds (Table 2). Also, 21-40-year-old homeless patients had a significantly (p=0.03) shorter time to be seen by an ED physician compared to the same age group of domiciled ED patients. Though marginally statistically significant (at alpha <0.1), when categorized by gender, homeless women had longer wait times than female domiciled patients. Semi-urgent (CTAS-4) homeless visit wait times were significantly longer (by 19 minutes at the median, p=0.0001) than domiciled patients in the same acuity level. Homeless patients who visited between 11pm and 7am (p=0.04) and also those who visited ED in the spring (p=0.04) waited longer. Homeless patients who visited ED during the morning hours (7am to 3pm) had significantly shorter wait times (p=0.005).

Comparison with CAEP clinical guidelines

CAEP position statement suggested targets are median wait times (time from triage to physician) of one hour and 90th percentile of three hours [25]. The study sample-based median wait times by CTAS-1-5 for this dataset were 14 (IQR=6-27), 60 (IQR=39-94), 119 (IQR=75-183), 81 (IQR=49-133), and 75 (IQR=43-119) minutes, respectively. CAEP criteria for the 50% benchmarks were met for CTAS-1-2. The 90th percentiles for this sample are 56, 156, 258, 199, and 176 minutes, respectively, and CAEP benchmark was met for CTAS-1-2 and CTAS-5.

In what follows, we explore the influence of homeless ED visits in meeting the abovementioned CAEP ED targets. By estimating the homeless patients' wait time coefficient (adjusted for age and gender) and using the statistical significance that compared homeless to domiciled patients of the same acuity level, we determine the influence of homeless visits. 

Factors Associated with 50th, 90th, and Above Three-Hour Wait Times

After adjustment for age and gender wait time differences, acuity level stratified analyses of wait time disparities are shown in Table 3. The median regression results, in model 1 (Table 3), by acuity level, which depicts at the 50% of visit benchmark, showed homelessness was significantly associated with waiting more than 20 minutes longer than domiciled and this was only at CTAS-4 level. The second regression model was fitted to the 90th percentile of visits to explore the factors associated with extreme wait times. Table 3 illustrates the beta coefficient for increased wait times in homeless compared to domiciled patients and the intercept. The intercept for each model (Table 3) illustrates the estimated wait time of the reference group of below 20-year-old, male, domiciled patients. The intercept was statistically significant (p<0.05) in each of the models. 

**Table 3:** Homeless wait time association, adjusted for age and gender CTAS: Canadian Triage and Acuity Scale; Non-sig: non-significant; coef: coefficient Model 1: 50th percentile regression. Model 2: 90th percentile regression. Model 3: Sample of patients who waited over the three-hour target p-value means significance level. Non-sig indicates a p-value >0.05 and * indicates a p-value <0.05 for the coefficient. All intercepts were statistically significant. Reference for homeless coefficient is domiciled

	Acuity level			
Factor	Emergent (CTAS-2)	Urgent (CTAS-3)	Semi-urgent (CTAS-4)	Non-urgent (CTAS-5)
Model 1				
Intercept	60 (58-62)	137 (134-139)	90 (88-92)	77 (73-81)
Homeless (coef)	Non-sig	Non-sig	20* (12-28)	Non-sig
Model 2				
Intercept	229 (205-253)	294 (289-299)	228 (223-233)	183 (171-195)
Homeless (coef)	Non-sig	88* (69-106)	91* ( 70-112)	127* (80-173)
Model 3				
Intercept	304 (285-322)	249 (246-252)	237 (70-112)	221 (233-241)
Homeless (coef)	-28* (-41--14)	40* (25-55)	31* (17-45)	57* (25-89)

The results showed homelessness was associated with more than an hour longer wait time (compared to domiciled), at all CTAS levels of 3-5, but has no association for CTAS-2 (model 2, Table 3). The third model illustrated factors associated with wait times of over 180 minutes. In that situation, homeless visits contributed to lengthening the wait time in all but CTAS-2 visits, in which homelessness was associated with wait times that were 28 minutes shorter.

The quantile regression results (Table 3) suggest that, after adjusting for age and gender differences, housing status was significantly associated with CTAS-4 wait time median (model 1, Table 3), 90th percentile (model 2, Table 3), and waiting longer than three hours (model 3, Table 3) for CTAS-3-5. Homeless patients consistently (across all model scenarios) waited longer than domiciled patients in CTAS-4 visits. Homelessness was the only significant predictor of 90th percentile wait time for CTAS-5 visits (other predictors are not shown in Table 3). Homeless visits were significantly associated with wait times greater than three hours (model 3, Table 3). 

Acuity level-based wait time disparity determining demographic subgroup identification

We used the CART method to identify the demographically classified subgroups associated with wait time disparities. The wait times noted in the figures given below are means for the age-gender-homelessness classified subgroups. The CART analysis results show the partitioning of wait time disparities to identify the demographic subgroups in which the discrepancies exist. In doing so, CART reveals the hierarchical structure of partitioning, starting from the most important demographic factor that demarcates the highest wait time disparity to the least important factor in the bottom. In the tree diagrams shown below, rectangular boxes indicate demographic groups starting from the most important (at the top) to the least important (at the bottom), lines show the subgroup classification, and hexagons give the mean wait time of the final subgroup. The final rectangles show the number in each of the final subgroups contributing to the last partitioning of wait times. The statistical significance noted in the final partition tests the hypothesis of the equivalence of the mean wait time of the final binary-classified subgroups using the p-value shown in the figure.

**Figure 2:**
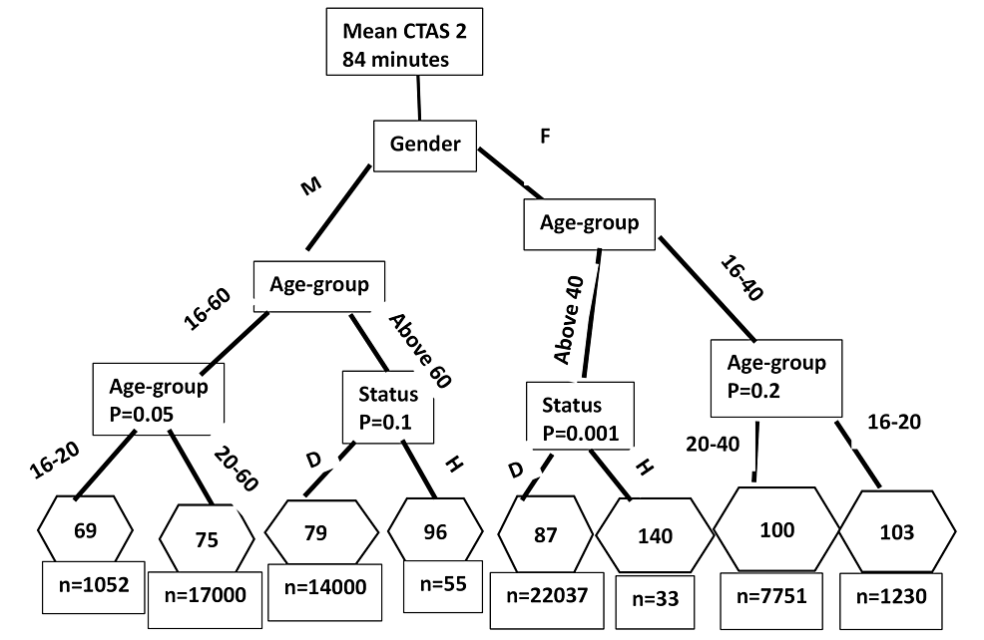
Emergent triage level (CTAS-2) demographic classification of wait time disparities CTAS: Canadian Triage and Acuity Scale; M: male; F: female; Status: housing status; D: domiciled; H: homeless; p: p-value The mean wait time (minutes) for the final classification node is included in the hexagon. p-value means significance level. The p-value tested the null hypothesis of equivalence of means in the final classification

For the CTAS-2 visits (Figure 2), gender was the most prominent wait time disparity classifier with females waiting longer than males (p<0.001). The next important CTAS-2 classifier was the age group, and above 40-year-old homeless females stayed the longest, 53 minutes longer than age-gender-matched domiciled patients (p=0.001), who stayed 87 minutes on average. Homeless 60-year-old men waited on average 20 minutes longer (p=0.1 statistically insignificant) than domiciled men of the same age group. No other significant housing status-related wait time disparities were found among CTAS-2 patient visits. 

**Figure 3:**
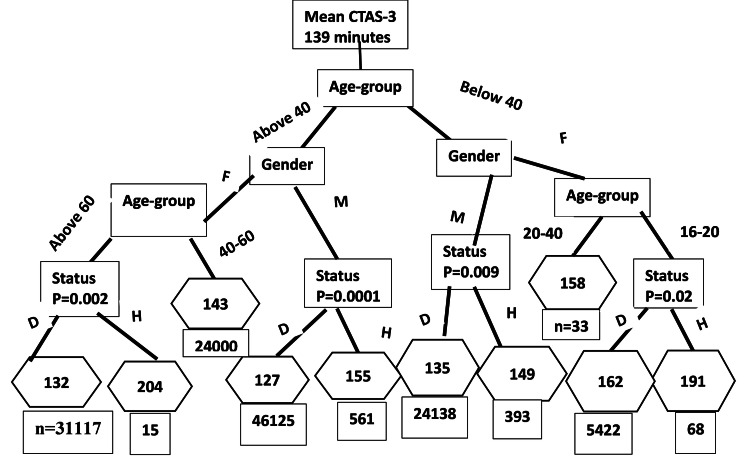
Urgent triage level (CTAS-3) demographic classification of wait time disparities CTAS: Canadian Triage and Acuity Scale; M: male; F: female; Status: housing status; D: domiciled; H: homeless; p: p-value The mean wait time (minutes) for the final classification node is included in the hexagon. p-value means significance level. The p-value tested the null hypothesis of equivalence of means in the final classification

For CTAS-3 patients (Figure 3), the most prominent determinant of wait time disparity was an age group cut-off of 40, followed by gender (females waiting longer than males), and then lastly the housing status demarcated the wait time discrepancy. The highest wait time discrepancy occurred between homeless females above 60 years of age, who stayed 72 minutes longer (p=0.002) than gender-age-group-matched domiciled patients, who stayed on average 132 minutes. Homeless females younger than 20 years of age waited 29 minutes (p=0.02) longer than age-gender-matched domiciled patients, who stayed 162 minutes, on average. Also, homeless males of age older than 40 waited 28 minutes (p=0.0001) longer than matched domiciled patients (mean wait time=127 minutes). No housing status-related wait time disparities were found within this triage level for 20-60-year-old females.

**Figure 4:**
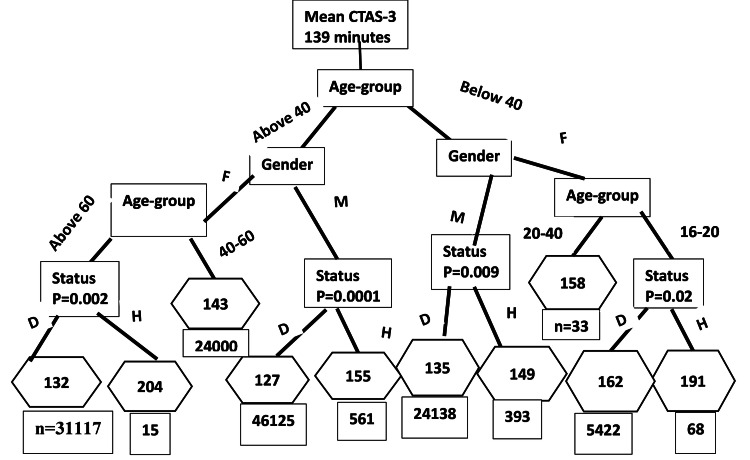
Urgent triage level (CTAS-4) demographic classification of wait time disparities CTAS: Canadian Triage and Acuity Scale; M: male; F: female; Status: housing status; D: domiciled; H: homeless; p: p-value The mean wait time (minutes) for the final classification node is included in the hexagon. p-value means significance level. The p-value tested the null hypothesis of equivalence of means in the final classification

For CTAS-4 (Figure 4) visits, gender was the most prominent wait time classifier, and for men, housing status was the second most prominent classifier. For women, age was the second most important classifier. Above 60-year-old homeless females had the longest mean wait time of 226 minutes (125 minutes longer than matched domiciled, p=0.0001), and the second longest waiting group for CTAS-4 level was homeless females in the age group of 40-60 (196 minutes). Homeless females above 60 waited 125 minutes longer (p=0.0001) and 40-60-year-old homeless females waited 95 minutes (p=0.0008) longer than age-gender-matched domiciled patients. No housing status-related wait time discrepancies were found for CTAS-4 level 16-20-year-old females, but 20-40-year-old homeless females waited 51 minutes longer (p=0.0001) than matched domiciled patients.

**Figure 5:**
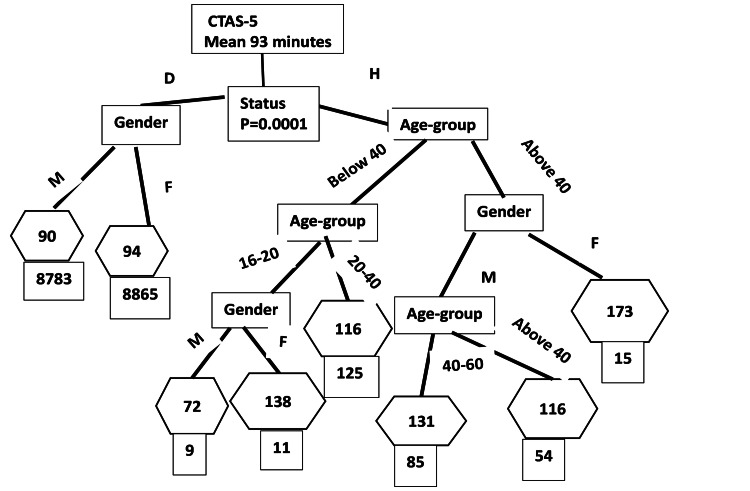
Non-urgent triage level (CTAS-5) demographic classification of wait time disparities CTAS: Canadian Triage and Acuity Scale; M: male; F: female; Status: housing status; D: domiciled; H: homeless; p: p-value The mean wait time (minutes) for the final classification node is included in the hexagon. p-value means significance level. The p-value tested the null hypothesis of equivalence of means in the final classification

Homelessness was the most prominent wait time-determining classifier for CTAS-5 visits (Figure 5). While EAP targets in this group were met, with 90th percentile of 176 minutes, and there were no notable age-gender-based disparities among the domiciled patients (who comprised 98.5% of these visits), homeless females older than 40 waited 82 minutes longer (p=0.0001) than age- and gender-matched domiciled patients. All of the other homeless age-gender subgroup wait time disparities for CTAS-5 level patient visits were either marginally significant or insignificant at the alpha level of 0.05. Homeless females younger than 40 stayed 21 minutes (p=0.04) longer than matched domiciled patients. Teenage homeless male patients waited a statistically insignificant 16 minutes less than (p=0.5) matched domiciled patients, while teenage homeless females stayed 39 minutes longer (p=0.09) than matched domiciled patients. Overall, homeless patients triaged as CTAS-5 waited 30 minutes longer (p=0.0001) than domiciled patients triaged as CTAS-5. Homeless male 16-20-year-olds waited the shortest time of 72 minutes for the triage level CTAS-5. 

Females over 40 with CTAS-2-4 visits were most vulnerable to prolonged waits, while in CTAS-5 visits, homelessness was associated with the longest waits. Longer waits for homeless females over 40 (n=520 visits) were consistent across all acuity levels compared to age-gender-matched domiciled patients.

## Discussion

The study site's ED visit data analyses revealed no acuity level-based discrepancies at the 50th benchmark between homeless and domiciled patients, with the exception of CTAS-4 visits. At the 90th percentile benchmark, however, an inconsistent pattern of equal length wait times in some homeless age-gender groups and longer in other groups was revealed. Based on these patterns, our findings do not support the notion that homeless patients are discriminated against in the ED by making them stay longer to receive care, as suggested in one US study [6]. Our data, being derived from an ED tracking system, provides little information beyond wait times and limited demographics, and more individual-level data would be required to ascertain the reasons for the wait time differences that we did find. Homeless patients with presenting conditions such as exacerbation of upper respiratory and flu-related illnesses, classified as low acuity levels CTAS-4 and CTAS-5, have the need for a warm shelter and thus have less inclinations to respond to the first calls to come in to see the ED physician to receive care, and this may result in prolong wait times recorded in the database for them. Anecdotal and research evidence [14,30] suggest that certain disruptive or intoxicated patients are often removed by law enforcement agencies soon after arrival or have assessment and treatment expedited by staff to minimize the disruption to the ED, potentially explaining the shorter wait times identified in 21-40-year-old and 16-20-year-old homeless patients.

Wait time disparities among subgroups of homeless female patients older than 40 were consistent across CTAS-2-5, with 40-76-minute longer waits compared to matched domiciled patients. Given that the percentage of homeless female visits in the age group above 40 was less than 0.2%, the impact of these longer wait times in this group on the 90th percentile of the wait time distribution is likely to be minimal. Further research may explain our findings more conclusively.

Our finding that homelessness is the most prominent wait time disparity classifier for non-urgent visits supports the theory that this population turns to the ED for their primary care needs and that enhanced access to primary care may both improve appropriate care and decrease the cost thereof. This finding also supports other Canadian research on the care of homeless people suggesting unmet healthcare needs, especially for mental health and substance use disorders [24] often represented in the CTAS-4 and CTAS-5 groups. We do not believe that our findings support the suggestion of US authors that longer ED wait times for homeless patients represent not the use of the ED for minor complaints but discrimination and bias against this population [6]. This was supported by our findings of inconsistent patterns of equal and less homeless wait times in some acuity levels compared to domiciled patients.

Further to the disclaim of discrimination in ED documented elsewhere [26,30], we found no differences in wait times for the median or 90th percentile between age-gender-matched homeless and domicile CTAS-2 level visits, suggesting equal response of staff regardless of housing status. We did find disparities in the mean wait time in homeless over 60-year-old men and women over 40, possibly reflecting a higher incidence of alcohol-related presentations, where staff may prefer to let patients "sober up" before bringing them in from the waiting room. Another potential reason for long waits for patients in this category may be the sympathetic inclination of staff to let patients spend longer in a warm waiting room, knowing that a more rapid transit through the ED puts the patient back on the street sooner. Although further subgroup-based research is needed to confirm these possibilities, confirmation thereof would support advocating for a sobering center and/or for enhanced shelter access.

We found disparities at the 90th percentile of wait times for homeless CTAS-3 patients and that homelessness was a significant predictor for waits longer than three hours (the top quartile of the wait time distribution). This discrepancy is possibly due to the same reasons suggested above.

Wait times for CTAS-4 level semi-urgent visits were significantly longer for the homeless compared to domiciled patients at the 50th and 90th benchmarks (Table 3). This discrepancy may be due to the subgroup of homeless males of all ages and above 20-year-old females waiting longer than age-gender-matched domiciled for this triage level. Longer wait time of 20-90 minutes that we found for homeless males of all ages and females over 20, again, suggests the need for enhanced primary care access. This demographic and acuity level characterization of homeless wait times are unique to this study; previous Canadian studies in other cities have identified homeless males visiting ED as having higher rates of psychiatric conditions and females as having higher rates of sexual assault-related visits [6,15], all which are categorized as CTAS-4.

The suggestion of primary care use of the ED is reinforced by the data for CTAS-5 level non-urgent visit wait time findings. Homeless females over 40, triaged to CTAS-5, waited 83 minutes longer than age-gender-CTAS-matched domiciled patients. Though unmet healthcare needs, lack of continuity of care, and personal- and system-level barriers to accessing primary care for the homeless are well documented in the literature [30], administrative data does not allow us to do a deeper evaluation of the influence of these factors.

Our findings from a single ED center visit wait time 50th percentile and 90th percentile benchmark deviated from the CAEP targets for low acuity triage level visits, more so among demographic groups of homeless, specifically homeless females and above 40-year-old adults.

Strengths and limitations

We provide the first Canadian tertiary care subgroup analysis of wait time disparities experienced by homeless ED patients. Using administrative data collected over five years, we identified demographic-level subgroups at risk of longer waits for different acuity complaints, pinpointing where CAEP benchmarks were not met. Our single-center data analyses disputed the common belief of long waiting in the ED by homeless patients due to discrimination.

Our analysis did not incorporate other factors contributing to wait time disparities such as presenting complaints within each triage acuity level or change of condition while waiting to be seen. The analysis does not include other factors contributing to lengthy wait times, such as volume, ED congestion, use and access to primary care, and output flow. Our findings came from a single ED site and may not be reflected in rural, non-teaching, or lower-volume ED, and future expanded research should confirm/dispute these findings using multiple ED in different settings. We acknowledge that there are many factors contributing to ED wait times than what we have considered in the analysis. These limitations should be considered as future areas of improvement. 

Implication for clinical practice, program planning, and policymaking

Despite the need for further work in this area, our findings do support the advocacy for alternative primary healthcare services for homeless people.

## Conclusions

Our findings coming from the largest ED in Atlantic Canada suggest homelessness was only an age-gender-related wait time disparity modifier in low acuity level ED visits. Homelessness-related wait time disparities exist in the low acuity level ED visits more than in the other levels, supporting the theory that lack of primary care access is a driver of ED use in this group. Our acuity level analysis supports that low acuity level triaged homeless people of a certain age (older) and gender groups (female) wait longer than their age-gender-matched domiciled patients to be seen by a physician. Overall, CAEP targets were met for CTAS-1-2 patients, regardless of housing status.

The CAEP wait time benchmark was met in high acuity level presentations only when unadjusted for age, gender, and homelessness-related variations. Homelessness was the prominent non-urgent level wait time discrepancy classifier, and if their non-urgent needs were met elsewhere, the excess wait time of 127 minutes can be reduced to meet the CAEP benchmark 90% of the time.
